# *MiR-422a* promotes loco-regional recurrence by targeting NT5E/CD73 in head and neck squamous cell carcinoma

**DOI:** 10.18632/oncotarget.9829

**Published:** 2016-06-04

**Authors:** Nathalie Bonnin, Emma Armandy, Julien Carras, Sylvain Ferrandon, Priscillia Battiston-Montagne, Marc Aubry, Sébastien Guihard, David Meyronet, Jean-Philippe Foy, Pierre Saintigny, Sonia Ledrappier, Alain Jung, Ruth Rimokh, Claire Rodriguez-Lafrasse, Delphine Poncet

**Affiliations:** ^1^ Hospices Civils de Lyon, Lyon, France; ^2^ EMR3738, Equipe de Radiobiologie Cellulaire et Moléculaire, Faculté de Médecine Lyon Sud - Charles Mérieux, Oullins, France; ^3^ Université de Lyon, Lyon, France; ^4^ Université Rennes 1, Université Européenne de Bretagne, Biosit, Faculté de Médecine, Rennes, France; ^5^ Plate-forme Génomique Environnementale & Humaine Biogenouest, Biosit/OSUR, Rennes, France; ^6^ CNRS, UMR 6290, Institut Génétique et Développement de Rennes, Rennes, France; ^7^ Laboratory for Tumor Biology and Tumor Bank Paul Strauss Cancer Center, EA3430 of The University of Strasbourg, Strasbourg, France; ^8^ “Stem Cell Transcriptomic Diversity’ Team, Cancer Research Center of Lyon (CRCL), INSERM 1052-CNRS 5286, Lyon, France; ^9^ Department of Translational Research and Innovation, CRCL, INSERM 1052-CNRS 5286, Lyon, France; ^10^ “Signalization Metabolism and Tumor Progression” Team, CRCL, INSERM 1052-CNRS 5286, Lyon, France; ^11^ Biochemistry Department, Transfer and Molecular Oncology Unit, South Lyon Hospital, Hospices Civils de Lyon, Pierre Bénite, Lyon, France

**Keywords:** HNSCC, CD73, miR-422a, personalized medicine

## Abstract

At the time of diagnosis, 60% of patients with head and neck squamous cell carcinoma (HNSCC) present tumors in an advanced stage (III-IV) of disease and 80% will relapse within the first two years post-treatment, due to their frequent radio(chemo)resistance. To identify new molecular targets and companion biomarkers, we have investigated the miRNome of 75 stage III-IV oropharynx tumors without relapse (R) or with loco-regional relapse (non-responder, NR) within two years post-treatment. Interestingly, *miR-422a* was significantly downregulated in NR tumors, in agreement with the increase in cell proliferation and adhesion induced by *miR-422a* inhibition *in vitro*. Furthermore, we identified *CD73/NT5E* oncogene as target of *miR-422a*. Indeed, modulation of the endogenous level of *miR-422a* inversely influences the expression and the enzymatic activity of CD73. Moreover, knocking down *CD73* mimics the effects of *miR-422a* upregulation. Importantly, in tumors, *miR-422a* and *CD73* expression levels are inversely correlated, and both are predictive of relapse free survival - especially considering loco(regional) recurrence - in vitro two independent cohorts of advanced oropharynx or HNSCC (N=255) tumors. In all, we reported, for the first time, that *MiR-422a* and its target CD73 are involved in early loco(regional) recurrence of HNSCC tumors and are new targets for personalized medicine.

## INTRODUCTION

Head and neck squamous cell carcinoma (HNSCC) is the sixth most common cancer worldwide. While tumors in stage I-II are curable, two thirds of patients have tumors in advanced stages (III-IV) at the time of diagnosis, and despite a treatment combining radio(chemo)therapy and surgery (when possible), nearly 80% will relapse within two years. As a result, the 5-year overall survival (OS) rate is currently under 50% [1]. More aggressive therapies, such as induction chemotherapy, carbon hadrontherapy, radical surgery or concomitant biotherapy, are available; however, their prescription without the support of predictive biomarkers has failed to improve survival but has increased toxicity [2–4]. Hence, the identification of reliable prognosis/predictive biomarkers has been a matter of intense research in the last decade. The initial strategy to prescribe intensified therapy to patients with a poor-predicted outcome. Meanwhile, dose de-escalation could be envisaged for patients with a good-predicted outcome to reduce the frequency of debilitating side effects (difficulties in swallowing, breathing, eating…), induced by the standard treatment. In this field, the Human Papilloma Virus (HPV) infection - known to be predictive of good outcome - is more and more considered as a marker for dose de-escalation, with encouraging results considering reduced toxicity (NCT01530997). However, treatment intensification based on HPV stratification may not be an adequate treatment strategy, since intensity-modulated radiation therapy (IMRT) improves the survival irrespective of the HPV status [5], and biomarker for dose intensification are awaited. Studies on functional biomarkers have highlighted the importance of biomarkers for hypoxia and positron emission tomography (PET) imaging, for prognosing, but they have so far not been routinely implemented in clinical applications. The difficulty is that HNSCC are heterogeneous tumors with different locations (oral cavity, oropharynx, nasopharynx, larynx and hypopharynx) and different biological histories and risk factors (viral infection, alcohol/tobacco intoxication…) (for review see [6]). Hence, dealing with the tumoral heterogeneity of these cancers is challenging. Recently, extensive genomic and transcriptomic (meta)analyses have characterized four [7] (revised to six [8]) molecular subclasses of HNSCC: Basal, Classical, Mesenchymal and Atypical [7]. This signature has paved the way for the development of personalized treatments, but cannot, as such, be translated into clinical routine tests. To conclude, regarding clinical practices, the challenge is no longer to identify global prognosis/predictive markers to prescribe intensified treatments based on standard chemo-radiotherapy approaches, but instead as Kang et al. proposed, to identify novel therapeutic targets and to develop predictive companion biomarkers [6].

MicroRNA (miRNA) is a good candidate for such a strategy, since it can be easily analyzed in biological fluids and can be directly targeted by innovative therapies. Indeed, 6 clinical trials evaluating an anti-miR-122 strategy are on-going in the context of hepatic diseases, and among them one has already entered phase II testing (Santis Pharma Corp.). Furthermore, the identification of microRNA targets could provide us with the opportunity to develop more conventional pharmacological approaches. Regarding the companion tests, these small RNA molecules are efficiently retrieved from (fixed or frozen) tumor samples or from biological fluids, such as blood, urine or saliva, and display high levels of stability over time and tissue specificity [9]. Four miR-based panels dedicated to the diagnosis of lung and kidney cancers are already commercialized for clinical use (Rosetta Genomics). With regards to HNSCC, microRNA signatures with a prognostic or a diagnostic significance have recently been identified, but need to be confirmed by conducting independent studies [10, 11]. Hence, the heterogeneity in tumor location is a limiting factor in conducting such molecular studies in the case of HNSCC. To circumvent such limitations, the present study proposes a two-step approach, in order to identify target/companion biomarkers for HNSCC treatment strategies. (i) We will initially identify potential target/companion biomarkers in a small yet highly homogenous cohort of patients, who will be selected according to a common criterion, namely the location of the HNSCC, before (ii) applying this personalized strategy to a larger HNSCC cohort encompassing different locations. In this study we have targeted the oropharynx, which is associated with a poor clinical outcome. While the frequency of hypopharynx and larynx tumors is decreasing, due to public policies against alcohol/tobacco consumption, the incidence of oropharynx tumors has gradually risen in the past two decades [12]. Most of the oropharynx tumors in advanced stage are radio(chemo)resistant and recur within the first two years post-treatment, giving rise to secondary cancers, metastases or loco-regional recurrences. Since the initial intra-tumoral context, which gives rise to secondary tumors, metastasis or to loco-regional recurrence is expected to differ, and could be a cause of molecular heterogeneity, we have included only tumors which have relapsed loco-regionally during the first two years post-treatment in our non-responder (NR) study group, while the responder (R) study group experienced no recurrence within the first two years. Overall, our objectives are (i) to identify a microRNA significantly deregulated in the NR group, (ii) to characterize its putative target(s), and (iii) to evaluate our companion marker/molecular target in a larger HNSCC cohort.

## RESULTS

### *MiR-422a* is significantly downregulated in oropharynx tumors from patients who experienced early loco(regional) recurrence

**Figure 1:**
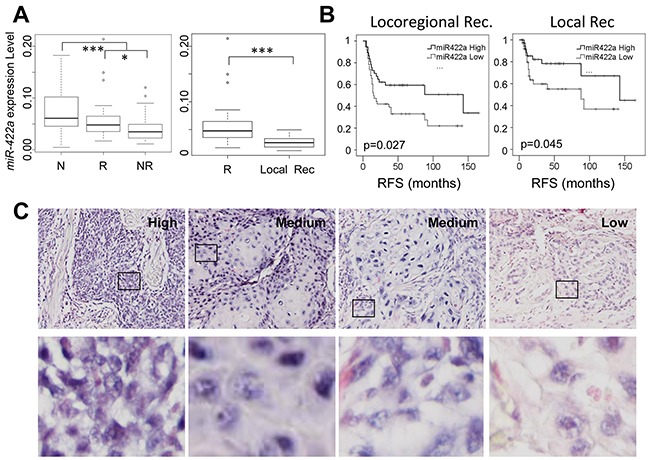
The level of expression of *miR-422a* is downregulated in oropharynx tumors from patients who have experienced early loco-regional recurrence **A.** The normalized expression level of *miR-422a* was determined on a TaqMan low density array (TLDA technology). *MiR-422a* is downregulated in tumor samples (R and NR) compared to healthy adjacent tissues (N), and in the NR versus the R subgroups (on the Left). It is especially downregulated in NR patients exclusively with local recurrence (Local Rec.) (On the Right). Wilcoxon tests were conducted, *p<0.05, **p<0.01, ***p<0.001. **B.** Kaplan Meier representations of Relapse Free Survival, considering loco-regional recurrence or only local recurrence (Right). **C.** Representative images (x200 magnification) of *miR-422a* localization by situ hybridization (purple labelling), on 4 samples with low, high or intermediate levels of *miR-422a* expression, as determined by RT-qPCR. An enhanced image of the outlined area (Bottom) shows an intense cytosolic and nuclear labelling in the high condition, and a faint cytosolic labelling in the “Low” condition, with intermediate levels in the “Medium” condition.

The level of expression of 384 miRNA was determined by RT-qPCR (TaqMan low density microarray) in 75 stage III-IV oropharynx tumors (36 from NR and 39 from R), and in 38 adjacent healthy tissues (N) (19 from NR and 19 from R). Overall, 13 miRNA were significantly deregulated in NR versus R (Wilcoxon test, p<0.05) and were also predictive of relapse-free survival (RFS) (LogRank test, p<0.05) (data not shown). We refined our initial analysis and searched for miRNA that could differentiate patients who exclusively experienced local (and not loco-regional) recurrence (Local Rec) from patients who did not recur (R). Among the 13 miRNA initially identified, only the downregulation of *miR-422a* was associated with an exclusively local recurrence (Figure 1A). Furthermore, the level of *miR-422a* expression was also predictive of RFS (LogRank test, p<0.05), when considering loco-regional or local relapse (Figure 1B). Regarding the healthy adjacent tissues (N), we observed a significantly higher level of *miR-422a* compared to tumor samples (Figure 1A, Left part). The RT-qPCR data were confirmed by conducting individual custom-made RT-qPCR experiments (Supplementary Figure S1) and by carrying out *in situ* hybridization (Figure 1C). Indeed, the intensity of the cytoplasmic and nuclear labelling of the cancer cells (and not of the stromal cells) increased as a function of *miR-422a* expression levels, determined by RT-qPCR (low, medium and high) in the same tumors (Figure 1C). Together with the high cellularity (over 70%) of our samples, this observation confirms a tumor-specific dysregulation of *miR-422a* expression.

### *MiR-422a* modulates cell adhesion and proliferation but not radio-sensitivity

**Figure 2:**
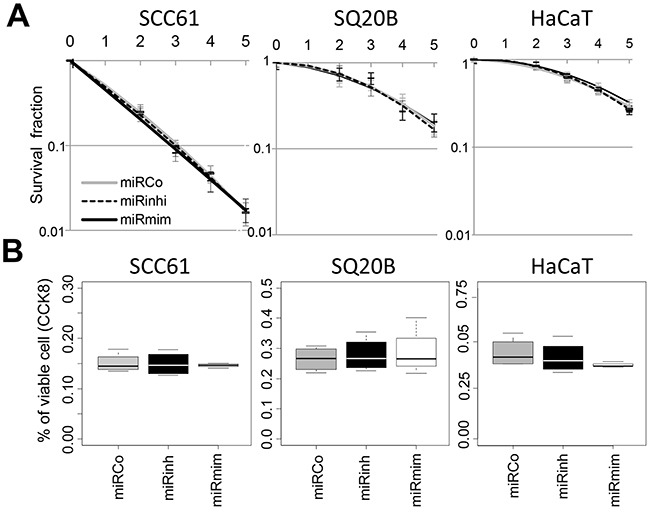
*MiR-422a* does not modulate cell response to irradiation **A.** Clonogenic assays were conducted on three cell lines transfected with either miRCo (control), miRmim (mimic) or miRinhi (inhibitor). The survival fraction is plotted as a function of the dose of X-rays irradiation. **B.** Cells were irradiated (10Gy) 24hr post-transfection, and cell viability was determined using the CCK8 assay 5 days after irradiation (percentage of the non-irradiated condition are shown). No significant statistical differences were observed in both experiments.

**Figure 3:**
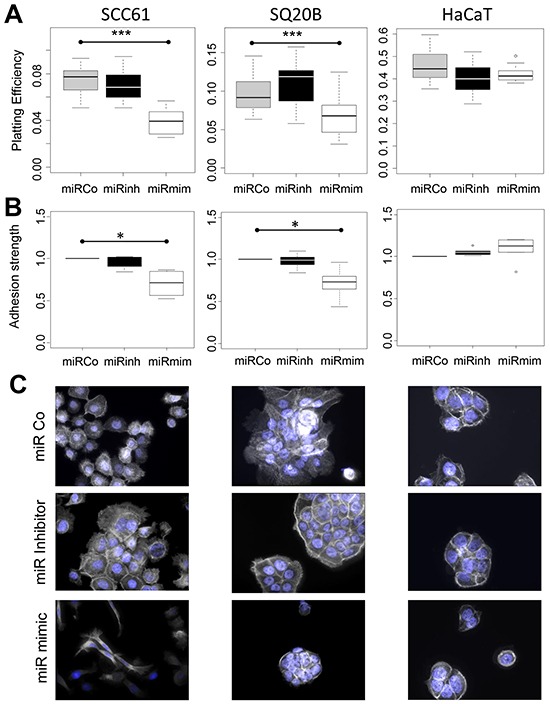
*MiR-422a* modulates adhesion of SCC61 and SQ20B cells **A.** Representation of the plating efficiency. Cells were transfected with the different constructs and plated at a low density. All of the clones were fixed and counted, once the 64-cell stage had been reached in the miRCo condition. For each condition the ratio between the number of clones and the number of plated cells is shown. **B.** The strength of adhesion (maximal impedance signal) was determined using the xCELLigence assay. Cells were transfected with the different constructs and plated, the signal was determined at full cellular confluence after 48 hr to 72 hr. **C.** Fluorescence imaging of actin cytoskeleton (white) and nucleus (blue) was conducted 48hr after transfection of the three cell lines (X40 magnification).

To better characterize the function of *miR-422a*, we conducted *in vitro* experiments on three cell lines: SCC61 and SQ20B derived from human HNSCC, as well as HaCaT derived from normal epithelial cells (all the three expressing *miR-422a* (Supplementary Figure S2)). Cells were transiently transfected with modified oligonucleotides, either mimicking (miRmim) or inhibiting (miRinhi) the endogenous expression of *miR-422a*. An irrelevant construct was used as a control (miRCo). The efficiency of the transfections was assessed using a fluorescent miRNA, and repeatedly reached 98% (data not shown). We initially evaluated whether the downregulation of *miR-422a* was responsible for radio-resistance (as local recurrence is indicative of radio-resistance). To do so, we carried out clonogenic assays (standard test for radiosensitivity measurement, Figure 2A) and analyzed cell viability using the CCK8 assay (Figure 2B), after transfection and irradiation, but we repeatedly failed to identify any effect of *miR-422a* modulation on cell sensitivity to X-ray irradiation. Strikingly, one parameter was constantly modified in the clonogenic assays, namely the plating efficiency (PE). The PE corresponds to the proportion of seeded cells able to produce a clone of at least 64 cells, after allowing sufficient time for 6 cellular divisions to occur in control cells. Hence, we observed a significant decrease in the PE in the miRmim versus the miRCo condition in SCC61 and SQ20B cells, but no modification was noted in HaCaT cells (Figure 3A). Different hypotheses can account for this decrease in the PE: (i) an increase in basal cell death, (ii) a decrease in the initial cell adhesion, and (iii) a decrease in cell proliferation, as previously reported [13, 14]. We first tested the level of spontaneous apoptosis, but observed no significant effect on the modulation of endogenous *miR-422a* levels in all the three cell lines (Supplementary Figure S3A). In order to determine the strength of adhesion of cells, we measured the maximal impedance signal (at time of full confluence) using the xCELLigence device. We noticed a significant reduction in the adhesive capacity of cells in the miRmim subgroup in SCC61 and SQ20B cell lines only (Figure 3B). Furthermore, the morphology and the actin cytoskeleton of these cells were altered (Figure 3C), since they displayed either a loss of (SCC61) or a disorganized (SQ20B) actin polymerization at the inter-cellular junctions in the miRmim condition. The resulting fibroblastic or round-shaped cells observed in the SCC61 and SQ20B, respectively, is concordant with a reduction in the strength of cell adhesion. Inversely, in the miRinhi condition, we noticed a more intense labelling at the inter-cellular junctions in the SCC61 and SQ20B cell lines. Moreover, no obvious modifications in actin polymerization or cell morphology were visible in the HaCaT cell line. Finally, we tested the consequence of *miR-422a* expression on cell proliferation in our cell lines, and conducted CCK8 assays (Supplementary Figure S3B). We confirmed an increase in cell proliferation in the miRinhi condition and a decrease in the miRmim condition. This effect was clearly visible in SCC61 and SQ20B cells, and less noticeable in the case of HaCaT cells (but still significant). These findings suggest that *miR-422a* induces a combined downregulation of cell adhesion and proliferation, at least in two of the cell lines studied.

### *MiR-422a* targets the oncogene *CD73/NT5E*

**Figure 4:**
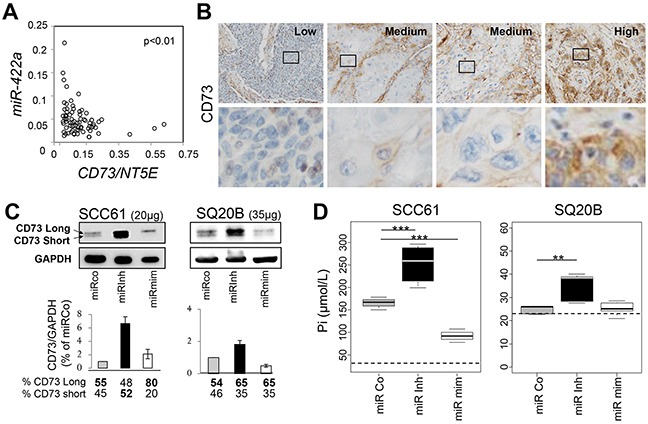
*MiR-422a* targets *NT5E/CD73* **A.** The *CD73* expression level was determined by RT-qPCR from the same samples as those used for measuring *miR-422a* expression levels, and was significantly inverse correlated with *miR-422a*. **B.** Representative images (x200 magnification) of immunohistochemically labelled CD73 (brown labelling), on the same samples used for ISH, with low, high or intermediate levels of expression of NT5E (and High, medium, and low levels of *miR-422a*, respectively, as determined by RT-qPCR). An enhancement of the outlined area is shown below, and reveals an intense membranous labelling in the “High CD73” (but “Low *miR-422a*”) condition, and a faint labelling in the “Low CD73” (but “High *miR-422a*”) condition, with intermediate levels in the “Medium” condition. **C.** Two days after transfection with the different miRNA constructs, cells were lysed and analyzed for CD73 and GAPDH content by Western blot analysis. The two isoforms (CD73 Long and short) are shown. The signal intensity was determined and normalized (GAPDH) for each isoform of CD73, the total intensity was set at 1 for the miRCo condition. **D.** Measurement of the enzymatic activity of CD73. Two days after transfection, cells were incubated with 2mM of AMP for 1 hr and the final concentration of the produced inorganic phosphate (Pi) was measured in the medium. The threshold for detecting Pi was determined and is represented by a dashed line. Wilcoxon tests were conducted, *p<0.05, **p<0.01, ***p<0.001.

Among the predicted targets of *miR-422a* by miR-map (http://mirmap.ezlab.org/app/) and miRecord (http://c1.accurascience.com/miRecords/), we searched for oncogenes known to modulate the adhesion and proliferation processes and tested their level of expression in our tumor samples. Among them, we focused on *CD73*. This ecto-5′-nucleotidase (also referred as *NT5E*) is a GPI-anchored cell surface protein, which plays enzymatic and non-enzymatic activities. Its oncogenic property partly relies on the modulation of cell adhesion and proliferation [15]. Interestingly, we demonstrated an inverse correlation (p<0.01) between the levels of expression of *miR-422a* and *CD73* (Figure 4A). Next, we confirmed this inverse correlation by analyzing an independent public dataset (Geoset GS33232), with expression data available for 44 HNSCC tumors and 25 normal tissues (from uvulo-palato-pharyngoplasty) (Supplementary Figure S4). Using IHC, we also observed an inverse correlation (Figure 4B) between the level of expression of *miR-422a* determined by RT-qPCR and the intensity of CD73 labelling (the IHC were done on the same four patients as for the ISH). Doing western-blot experiments in basal condition, we barely detected CD73 in the HaCaT cell line (Supplementary Figure S5), we thus focused the subsequent experiments exclusively on the SCC61 and SQ20B cell lines. Thereby, we observed two bands corresponding to the very recently described isoforms of CD73: the full-length isoform 1 (CD73L, 63kDa) and the short isoform 2 (CD73S, 58 kDa), deprived of the catalytic domain. Isoform 2 is believed to be specifically deregulated in cancers [16]. As expected, we noticed a clear increase in the total amount of CD73 after the inhibition of endogenous *miR-422a* (miRinhi, Figure 4C) and a decrease (at least in the short isoform) in miRmim in the two cell lines. Next, we measured the enzymatic activity of CD73 in SCC61 cells, and confirmed a clear increase in the case of miRinhi and a decrease in the miRmim condition (Figure 4D). The effect was also visible in the miRinhi condition in SQ20B cells, despite a ten-fold lower basal activity (in relation with a much lower basal protein level). To conclude, miR-422a directly impacts the mRNA and protein level of CD73, as well as its enzymatic activity.

**Figure 5:**
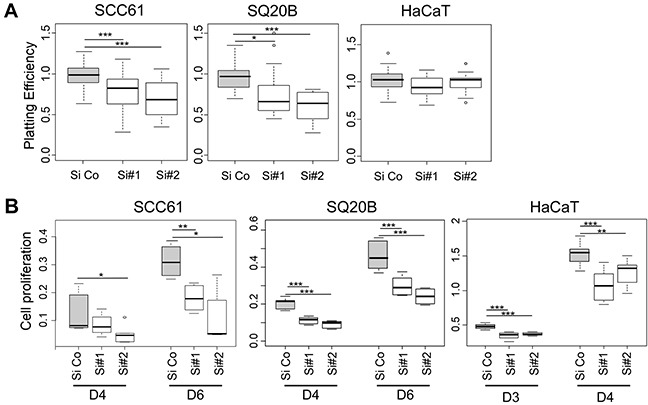
The silencing of *CD73* phenocopies *miR-422a* mimic effect The three cell lines were transfected with an irrelevant siRNA (Si Co) or with two siRNA targeting *CD73* (Si#1 and Si#2), and plated in different conditions. **A.** Determination of the plating efficiency. **B.** Quantification of cell proliferation using the CCK8 assays at days 4 and 6 post-transfection. The luminescent signal is normalized for 1 000 seeded cells. Wilcoxon tests were conducted, *p<0.05, **p<0.01, ***p<0.001.

To confirm the implication of CD73 on cell adhesion and proliferation in our cell lines, we conducted *CD73* knocked-down experiments. First, we validated the efficiency of ours siRNAs at the protein and functional levels (Supplementary Figure S5). Next, we inquired the adhesive function of CD73 and as expected, our results confirmed a decrease in the PE in SCC61 and SQ20B cell lines, but not in HaCaT cells, after CD73 knocking down (Figure 5A). Furthermore, a decrease in cell proliferation was clearly noted in all three cell lines treated by siRNA targeting CD73 (Figure 5B). Regarding the cell morphology, CD73 was recently shown to promote cortical actin polymerization and to increase the membranous localization of E-cadherin, β-catenin, and Na+K+ ATPase, thereby preserving epithelial integrity [17]. This is in agreement with our observation with regards to an increase in cortical actin polymerization in the miRinhi condition (Figure 3C). To go further, we also noted an increase in membranous E-cadherin labelling in SCC61 and SQ20B cell lines treated by miRinh (Supplementary Figure S6).

To summarize, miR-422a and CD73 have the inverse effects on cortical actin polymerization, and on cell adhesion and proliferation.

### Why does CD73/*miR-422a* modulate cell adhesion and proliferation in a cell-type-specific manner?

Next, we investigated the differential effect observed on cell adhesion in SCC61 and SQ20B cells versus HaCaT cells. We observed a ten-fold reduction in the level of *CD73* mRNA in SQ20B and HaCaT cells (Supplementary Figure S2), which resulted in similar basal enzymatic activities (Supplementary Figure S5). Since the CD73 protein was hardly detected on Western blots of HaCaT cells, compared to SQ20B cells, this suggests (i) that an additional post-translational control of CD73 exists in HaCaT cells, and (ii) that most of the CD73 protein is dedicated to enzymatic functions in HaCaT cells. As adhesion is believed to be controlled by the non-enzymatic activity of CD73 [18, 19], we propose that the residual CD73 protein detected in HaCaT cells may play a minor role in cellular adhesion. However, HaCaT cells have a much stronger basal adhesion than SCC61 and SQ20B cells (Supplementary Figure S2), thus indicating that this function mainly relies on a different pathway in this cell line. Altogether, these findings explain the absence of measurable effect of *miR-422a*/*CD73* modulation on cell adhesion, despite a slight effect on cell proliferation, specifically reported in the HaCaT cell line.

### A high level of *CD73* expression is associated with early loco(regional) recurrence in two independent cohorts of patients suffering from HNSCC

In the ensuing set of experiments, we set the median expression value of *CD73* (and *miR-422a*) as a threshold to separate patients into “high CD73” or “low CD73” subgroups in our oropharynx cohort such as in the TCGA cohort.

**Figure 6:**
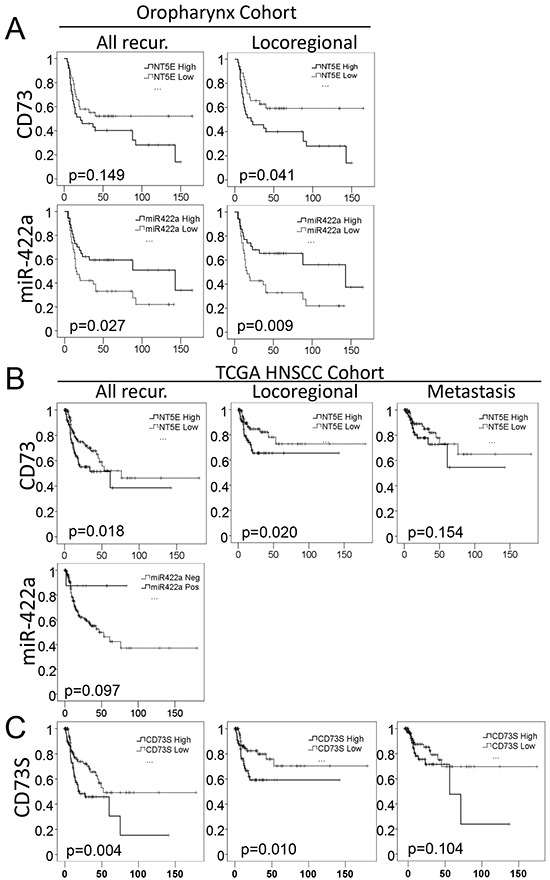
*MiR-422a* and *CD73* expression levels are predictive of loco(regional) recurrence in two independent cohorts **A.** RFS for all of the patients (All Recur) of our oropharynx cohort, or for patients without metastasis at the time of loco-regional recurrence (Locoregional) from the same cohort. **B-C.** RFS was extracted from the TCGA database, for patients suffering from head and neck tumors (not restricted to oropharynx), at stages III and IV (N=255). Kaplan Meier representation of RFS as a function of total CD73 (B) or CD73S (C) for all types of recurrences (on the Left) or restricted to loco-regional (Middle) or metastatic (Right) recurrences (as first event). LogRank tests were conducted to calculate the p-values.

Regarding the oropharynx cohort, we confirmed a shorter RFS in the “high CD73” subgroup (All recur., Figure 6A), although this correlation was not significant (p=0.149). Since we demonstrated (above) that CD73 increases the strength of cell adhesion, we speculated that its levels may be lower in tumors with an inclination to produce metastasis. We thus refined our RFS analysis by removing five patients who concomitantly displayed loco-regional recurrence and metastasis (Locoregional, Figure 6A), and observed a significant predictive effect of *CD73* expression level on RFS (with an even higher predictive value for *miR-422a*) (Figure 6A). It is worth noting that due to our initial inclusion criterion (half NR, half R), our cohort is biased towards a higher proportion of responder patients. Since target therapies have been much-awaited for treating patients who are in advanced stage of the disease, irrespective of the initial metastatic status or tumor localization, it was thus necessary to confirm our observations on a non-biased independent cohort of stage III-IV HNSCC tumors.

To do so, we extracted expression data from the TCGA database for 255 patients with stage III-IV tumors of different locations (oral cavity, oropharynx, larynx and pharynx). We observed a significant (p=0.018) increase in the risk of recurrence in the “high CD73” subgroup (Figure 6B, All recur.). The significance is maintained when only loco-regional recurrence is included (p=0.020), but is lost when only considering metastasis (p=0.154), as first event of recurrence. This strengthens the hypothesis that a high expression of *CD73* favors loco-regional recurrence, but may be deleterious for the metastatic process. Using the same TCGA database, *miR-422a* expression level was available for 465 samples (of which 42 healthy tissues and 423 HNSCC tissues). However, only 5.8% of the samples were positive for *miR-422a* expression (maybe due to a lower sensitivity of the technic used), though a lower proportion of positive samples was observed in the tumors (4.7%) versus healthy tissues (16.7%), what confirmed our result. Selecting the stage III-IV patients (N=205) and despite a very low number of *miR-422a* positive specimens (N=9), we drew the RFS curves and observed a clear tendency (obviously not significant) to a longer survival time without recurrence (Figure 6B). Hence, a biotherapy targeting *miR-422a*/CD73 may be highly beneficial for patients displaying *miR-422a*Low/*CD73*high tumors, to prevent loco-regional recurrence in HNSCC in advanced stages (all locations included).

CD73S, the splice variant of CD73L, is expected to bear the same 3′UTR sequence, comprising the *miR-422a* binding sites. Since CD73S is presumed to play a major role during oncogenesis [16], we investigated its expression in tumors from the TCGA database and observed a linear correlation between the transcript levels of CD73S and CD73L (in agreement with a co-regulation of both transcripts by *miR-422a*) (Supplementary Figure S8). Moreover CD73S is predictive of RFS considering all types of recurrences (p=0.004) or loco-regional recurrences (p=0.010), but is not predictive of metastasis (p=0.104) (Figure 6C). To conclude, *miR-422a* modulates the expression of both CD73S and CD73L, and CD73S bears the same predictive value (with even better p-value) with regards to RFS in Head and Neck tumors as compared with total CD73.

Regarding the influence of the clinico-pathological criteria by Cox modeling, we report a significant effect of the resection quality (with a higher risk of recurrence after incomplete resection as expected) in both cohorts (Table 1). We also determined the HPV status in our oropharynx tumors but failed to note any significant effect of this criterion, in both cohorts. This may either be due to a misdetection of the virus or to a low percentage of HPV-positive tumors in the two cohorts (oropharynx cohort 13% - N=75, TCGA 27% - N=67). Nevertheless, specific studies addressing the microRNA signature associated with the HPV status in oropharynx did not retrieve *miR-422a* [20].

**Table 1:** Influence of clinical parameter on RFS

	Oropharynx stage III-IV		HNSCC stage III IV (TCGA)	
	HR (CI)	p	HR (CI)	p
Gender	0.83 (0.37 - 1.86)	0.646	0.89 (0.5 - 1.58)	0.716
Age	1 (0.97 - 1.03)	0.901	1 (0.63 - 1.59)	0.998
Stage	2.38 (0.92 - 6.12)	0.073	1.58 (0.91 - 2.75)	0.106
Margin	1.59 (1.14 - 2.23)	0.007 **	1.54 (1.18 - 2)	0.001**
Alcohol/tobacco	0.8 (0.24 - 2.61)	0.708	1.01 (0.64 - 1.61)	0.963
HPV	0.43 (0.13 - 1.41)	0.165	0.36 (0.05 - 2.74)	0.327
*NT5E*	0.64 (0.35 - 1.18)	0.151	0.6 (0.38 - 0.94)	0.027*
*MiR-422a*	1.99 (1.07 - 3.7)	0.030 *	3.03 (0.42 - 21.93)	0.271

## DISCUSSION

In the present study we have demonstrated that *miR-422a* expression is significantly downregulated in oropharynx tumors from patients who have experienced early loco(regional) recurrence, as well as in tumor compared to normal tissues. *MiR-422a* downregulation is associated with earlier recurrence *in vivo*, which may be linked with the stronger cellular adhesion and higher rate of proliferation observed *in vitro*. We have shown that the mRNA, protein and enzymatic activity of the CD73 nucleotidase, which is involved in the oncogenic processes, are modulated by *miR-422a*, and that *CD73* downregulation mimics the effects of *miR-422a* overexpression. Furthermore, we have proven the predictive value of *miR-422a*/*CD73* regarding the loco-regional recurrence of stage III-IV tumors in our oropharynx cohort, as well as in the TCGA cohort encompassing different tumor locations. From these observations, we conclude that *miR-422a* downregulation promotes local recurrence in stage III-IV HNSCC, by targeting CD73.

There is a growing body of evidence strengthening the anti-oncomiR function of *miR-422a*. Its downregulation in tumoral versus normal tissue has been reported in osteosarcoma [21], in colorectal cancer [13, 22], hepatocarcinoma [14] and gastric tumors [23]. A low expression of *miR-422a* is a marker of poor prognosis in osteosarcoma [21] and colorectal cancer [24] and of poor RFS in hepatocarcinoma [25]. It has also been associated with chemo-resistance in osteosarcoma [26]. Its level of expression in tumors and in serum have been significantly correlated [25], paving the way for its establishment as a companion test. Salivary miRNA profiling has been developed for oral cancer [27]. This non-invasive, rapid and straightforward technique for assessing the expression of miRNA in the vicinity of the tumor, will be the next step in validating the use of *miR-422a* as a companion biomarker in saliva.

With regards to the function of *miR-422a*, its anti-proliferative effect has been reported in other cancer cell lines [13, 14]. Zhang et al. demonstrated its ability to inhibit cell proliferation and migration *in vitro*, as well as tumor growth and metastasis *in vivo*, in hepatocarcinoma [14]. They identified FOXG1 as the target of *miR-422a* responsible for these effects in hepatocarcinoma and demonstrated a direct regulation of*miR-422a* expression by FOXG1 (we only observed a faint regulation of *miR-422a* expression by our target (Supplementary Figure S7)). FOXG1 was part of our initial screening but its expression was not modified by *miR-422a* modulation in our model (data not shown). Instead, we identified a novel target of *miR-422a*, namely CD73. *NT5E/CD73* is an oncogene, which is involved in enzymatic and non-enzymatic activities. As an enzyme, its ecto-5′-nucleotidase activity is responsible for hydrolyzing ADP into phosphate and adenosine, which in turn initiates adenosine receptor-dependent signals, or is imported into the cell by nucleoside transporters [15]. The adenosine receptor-dependent signaling is used by tumoral cells that overexpress *CD73*, to facilitate their immune escape [28, 29]. However, CD73 is also a signaling and an adhesion [17, 18] protein involved in metastasis [30, 31], neovascularization[32] and tumor promotion [33], independently of its immune functions. Its overexpression has already been implicated in the resistance to treatment *in vitro* [34] and *in vivo* [33].

Regarding the clinical aspects, *CD73* overexpression is predictive of a poor prognosis in colorectal, gastric, gallbladder and triple negative breast cancers, as well as in chronic lymphocytic leukemia (for review see [15]). Overall, our results corroborate the pro-adhesive and pro-proliferative functions of CD73, along with its involvement in treatment resistance. However, in our study a high level of *CD73* appeared to be unrelated to the metastatic capacity in our cohort and in the TCGA dataset. Consistently, the results published in the literature are conflictual about the pro-migratory and the pro-metastatic function of CD73. In meduloblastoma cell lines, the metastatic potential is inversely correlated with *CD73* expression and activity [35]. Pharmacological inhibition of CD73 reduces adhesion but increases the invasion/migration capacities *in vitro* [17, 36], and treatment with adenosine (produced by CD73) inhibits cell migration and invasion [37]. More recently, loss of CD73 has been involved in epithelial barrier misstructuration and endometrial tumor progression [17].

One plausible explanation of these controversial observations is that numerous factors can influence the consequence of *CD73* upregulation, (i) the interaction with the host immune function *in vivo*, (ii) its participation in enzymatic activities versus its function in adhesion/signaling, (iii) the destination of adenosine: receptor fixation (while A1, A2A and A2B are pro-proliferative, A3 plays pro-apoptotic functions [38]), or intracellular uptake (which inhibits cell growth and favors apoptosis [39]), (iv) the intra-tumoral cellular subtype expressing CD73 [40, 41], (v) and the isoform that is upregulated. Considering the latter, it was recently shown that the short isoform, CD73S, is responsible for the pro-proliferative activity of CD73 [16] and for the tumor aggressiveness. This is in agreement with the higher significance of the predictive value on RFS of CD73S versus total CD73. CD73S, devoid of its catalytic domain, heterodimerizes with CD73L and inhibits its nucleotidase activity (partly by targeting it to the proteasome). This may explain the increase in the CD73L/CD73S proportion in the miR-mimic condition in SCC61 cells (Figure 5D). Knocking down the short isoform alleviates the proteasomal degradation of CD73L, which results in the paradoxical increase in CD73L and in an inverse proportion of the two isoforms. With regards to the influence of the cellular sub-population (stromal or cancerous cells) overexpressing CD73 inside the tumor, an increase in the stroma associated with a decrease in the tumoral cells, is associated with a good prognosis in rectal adenocarcinoma [41]. Considering our IHC observations, we also report a differential labelling of the stroma and the tumor for CD73 (Figure 4B), which is in agreement with this publication. Taken together, CD73 is a bi-functional protein, which shares both pro- and anti-oncogenic properties depending on multiple factors.

Targeting the adenosine receptor by anti-A2A drug has reversed the resistance to doxorubicin in mice model of triple negative breast cancer model [33]. However, blocking adenosine receptor only prevents a small part of the oncogenic effect of CD73, and therapeutic antibodies or pharmacological approaches targeting CD73 seem more appropriate to reach a higher therapeutic efficiency. These strategies have demonstrated anti-tumor activity in various xenograft mice models of breast, ovarian, colon and prostate cancers [30, 31, 42–44] and are now being developed for humans [45, 46]. Indeed, the anti-CD73 monoclonal antibody MEDI9447 targets and binds to CD73, leading to its clustering and its internalization, abolishing its activity. This immunotherapy combined with anti-PD-L1 is currently under evaluation in phase I clinical trials, for adult solid tumor (NCT02503774). It is thus reasonable to think that HNSCC patients in advanced disease stages with a high intra-tumoral level of CD73 may benefit from MEDI9447 in a near future. However, these drugs should be carefully prescribed taking into account the above mentioned considerations and the immune function of CD73. Furthermore, such an administration should be combined with a treatment preventing metastasis. The targeting of *miR-422a* is also plausible, with innovative therapies targeting microRNA currently being evaluated in clinical trials for different diseases. The drugs available so far can either mimic the microRNA (proMir) or inhibit it (antiMir). For example, the MiRagen Corporation developed a miR-29b mimicking strategy to treat fibrosis, and following the successful preclinical assays, the company is now entering phase I of clinical trials. Regarding the antiMir strategy, Miravirsen a drug dedicated to the treatment of hepatitis C, developed by Santaris Pharma, has completed a phase 2 trials. In the near future, targeting *miR-422a* with this kind of molecules could represent a good therapeutic strategy for treating patients with an unfavorable-predicted response to standard radio(chemo)therapy, on the basis of their *miR-422a* value.

## MATERIALS AND METHODS

### Clinical specimens

Fresh frozen samples of oropharynx stage III- IV tumors were obtained from the CRLCC Paul Strauss Center Tumor Bank (Strasbourg, France). Written informed consent was obtained from all patients and the study was approved by the local Committee on Human Research. Clinico-pathological and follow-up information were available for all patients (Table 2). Patients had been treated by surgery (the sample are pieces of the surgical resection) and radiotherapy (with or without chemotherapy). Only tissues with high cellularity (over 70%) were included. Patients were all negative for metastasis at the time of diagnosis. Patients who did not relapse during the first two years after surgery and radio(chemo)therapy, were considered as responders (R), while patients who experienced local (head and neck localization) and/or regional (nodes involvement) recurrence as a first event, within two years, were considered as non-responders (NR); patients with metastasis as a first event were excluded from the NR group.

**Table 2:** Clinical characteristics of patients

	NR		R		Total	
	No.	(%)	No.	(%)	No.	(%)
	36	(48)	39	(52)	75	(100)
Sex						
Male	30	(83)	31	(79)		
Female	6	(17)	8	(21)		
Age	Median	(range)	Median	(range)	Median	(range)
	56	(39-74)	53	(40-82)	54	(39-82)
Alcohol and tobacco use						
Yes	29	(81)	30	(77)	59	(79)
No	3	(8)	3	(8)	6	(8)
NA	4	(11)	6	(15)	10	(13)
HPV status^*^						
Positive	3	(8)	7	(18)	10	(13)
Negative	33	(92)	32	(82)	65	(87)
Disease site						
Tonsil	9	(25)	13	(33)	22	(29)
Base of tongue	10	(28)	14	(36)	24	(32)
Soft palate	7	(19)	4	(10)	11	(15)
Pharyngeal wall	1	(3)	3	(8)	4	(5)
Vallecula	5	(14)	4	(10)	9	(12)
other	4	(11)	1	(3)	5	(7)
Staging						
III	4	(11)	9	(23)	13	(17)
IV	32	(89)	30	(77)	62	(83)
N0	3	(8)	6	(15)	9	(12)
N1	4	(11)	7	(18)	11	(14)
N2	22	(61)	23	(59)	45	(57)
N3	7	(19)	3	(8)	10	(13)
Treatment						
RT alone	28	(78)	30	(77)	58	(77)
RT-CT	8	(22)	9	(23)	17	(23)

* Sample positive for viral DNA and RNA, RT: radiotherapy, RT-CT: radio(chemo)therapy, NA: not available.

### Data mining from TCGA and GEOset databases

We queried The Cancer Genome Atlas (TCGA) data portal and the Global Environmental Outlook (GEO) data portal (http://www.ncbi.nlm.nih.gov/geo). To test for an inverse correlation in the expression levels of *miR-422a* and *CD73*, we used the GSE33232 series including 44 HNSCC and 25 healthy tissues from uvulo-palato-pharyngoplasty. To assess relapse-free survival (RFS), we used the ‘TCGA2STAT’ R package [47] to download RNAseqV2 normalized read counts (RPKM), as well as miRNASeq normalized read counts (RPMM). Both expression and clinical data were available for 473 HNSCC tumors (from different locations: oral cavity, oropharynx, larynx, and pharynx). RFS for stage III-IV tumors (with or without metastasis at the time of diagnosis) was available for 255 patients; among them, 179 did not relapse, 40 relapsed loco-regionally, 31 developed metastasis and 4 developed another cancer, as a first relapse event.

### Molecular biology

Total RNA was extracted by using miRNeasy Mini Kit (Qiagen); quality was controlled by the small RNA kit for the Agilent Bioanalyzer (Bio-Rad). 0.7μg of total RNA were reverse transcribed and analyzed using the TaqMan Low density (TLDA) technology (human microRNA panel V2.0) as previously published [48]. The expression data (delta 2 CT method) were normalized against the geometrical mean of three reference genes (*Let.7a, miR-26a, Let.7e*) chosen according to the GeNorm instructions (https://genorm.cmgg.be/). Custom RT-qPCR were conducted on *miR-422a* and the three reference genes using specific TaqMan® MicroRNA Assays and the same master mix, on a MxPro 3000 (Agilent). *In situ* hybridization (ISH) against *miR-422a* was carried out following the instructions of the miRCURY LNA microRNA ISH Optimization kit (Exiqon). Regarding *CD73* quantification, 0.5 μg of total RNA were retro-transcribed using the QantiTect RT kit and qPCR were prepared using the QuantiTect SYBR® Green PCR Kit (ThermoFischer Scientific). Primers were designed to amplify the *CD73* isoforms 1 and 2 (Fd : TTATTCGACTGGGACATTCG-3′, Rs : 5′-AGGC CTGGACTACAGGAACC-3′), the *CD73* isoform 2 (Fd: 5′-TGATGAACGCAACAATGGAAT-3′, Rs:5′-TCTGGAACCCATCTCCACCA-3′), the TATA-box binding protein (*TBP*) (Fd : 5′-TATAATCCCAAG CGGTTTGC-3′, Rs :5′-CACAGCTCCCCACCATATTC -3′), the ribosomal protein L19 (*RPL19*) (Fd :5′-GGCACATGGGCATAGGTAAG-3′, Rs : 5′-CCATG AGAATCCGCTTGTTT-3′) and the glyceraldehyde-3-phosphate dehydrogenase (*GAPDH*) (Fd : 5′ GAGTCA ACGGATTTGGTCGT-3′, Rs : 5′- TTGATTTTGGAGGG ATCTCG-3′). The level of expression was normalized against the geometrical mean of three reference genes (*RPL19*, *TBP*, and *GAPDH*).

### Cell viability and proliferation

HaCaT, SQ20B and SCC61 cell lines derived from human normal epithelium, larynx and oropharynx tumors, respectively, were cultured as described previously [49]. MirVana microRNA Mimics (ID: MC12541) and Inhibitors (ID: MH12541) specific for *miR-422a*, or a Mimic negative control (Ref: 4464058) were transfected under the following conditions for 100 000 cells: 6.7 μL of Hiperfect (Qiagen), 0.3 nmol of oligonucleotides in 500 μL of final complete medium. Clonogenic assays were conducted as previously described [50]. The surviving fraction was calculated using the formula S(D)= n(D)/PE.N(D) where n is the number of colonies, N the seeded-cell number for a given dose (D) and PE the plating efficiency (PE= n/N, at 0 Gy). Apoptosis was measured 48 hrs post-transfection, using the Annexin-V and propidium iodide Apoptosis Detection Kit APOAF (Sigma-Aldrich), on an LSRII (Beckman-Coulter) flow cytometer. The number of viable cells was determined by measuring their metabolic activity using the Cell Counting Kit-8 (CCK8) (Sigma-Aldrich). Cells were seeded at different concentrations after transfection in a 96-well plate (4 wells per condition), and cell number was evaluated using the CCK8 on a Luminoskan Ascent Microplate Luminometer (Thermo-Scientific) at day 4 and 6 post-transfection, according to the manufacturer's protocol. Signal (Arbitrary Unit) was normalized for 1 000 seeded cells.

### Cell adhesion

To assess the strength of cellular adhesion, cells were seeded after transfection onto a 16-well E-plate (4 wells per condition), and impedance was measured for 3 days on the xCELLigence RTCA DP device (Ozyme). The maximum signal intensity reached at the time of maximum cell confluence, was set as the adhesion strength. Microscopic imaging was conducted two days post-transfection. Cells were fixed and stained as previously described [49]. The actin cytoskeleton was labelled using phalloidin-FITC (Sigma-Aldrich), and the DNA using Hoechst 33258 (Sigma-Aldrich). Observations were done on an Imager Z2 (Zeiss).

### Determination of CD73 activity and protein level

Cells were transfected with Silencer Selected siRNA (Life Technologies): one control and two targeting CD73 (ID: s9735#2; s9734#1), under the following conditions for 100 000 cells: 6.7 μL of hiperfect, 12.5 pmol of siRNA in 500 μL of final complete medium. Two days after transfection, cells were lysed and protein extracted and analyzed by western-blotting as previously published [49]. Anti-CD73 (ab124525, Abcam) or anti-GAPDH (H86504M, Interchim) were used as primary antibodies and HRP-linked goat anti-rabbit (sc3837, SantaCruz) or goat anti-mouse (sc2031, SantaCruz) as secondary antibodies. Immunohistochemistry (IHC) against CD73 was performed using a Ventana Autostainer Automat (Ventana Medical Systems). Slides prepared from formalin-fixed paraffin-embedded tumor specimens were incubated with an anti-CD73 antibody (ab124525, Abcam; dilution: 1/100). Signals were detected using the ultraView Universal DAB Detection Kit (Ventana Medical Systems), according to the manufacturer's instructions. Nucleotidase activity was determined as previously published [51]. Briefly, after three washes, cells were incubated for 1 to 3 hrs in a medium containing 2mM ATP. The concentration in inorganic phosphate of the supernatant was then analyzed using the malachite green colorimetric reaction. Standard dilutions of KH_2_PO_4_ solution (10 to 100 mM) were used to calculate the inorganic phosphate concentration.

### Statistical analyses

Survival analyses were performed with the R survival package software (R software: Language and Environment for Statistical Computing, R Foundation for Statistical Computing, http://www.R-project.org). Cox proportional hazard models provided estimates of the hazard ratios (HRs). For each covariate, we have checked that no evidence of violation of the proportional hazards assumption was found by plotting the scaled Schoenfeld residuals generated by the cox.zph function). A Wilcoxon test was used for mean comparison, while linear correlations were conducted using the spearman test. Considering the relapse free survival analysis: LogRank and Kaplan Meier curve drawings, were done using the SPSS software (IBM, France).

## SUPPLEMENTARY FIGURES


