# Supplementary material for: MiR-422a promotes loco-regional recurrence by targeting NT5E/CD73 in head and neck squamous cell carcinoma
Source: Oncotarget. 2016 Jun 4;7(28):44023–38. doi: 10.18632/oncotarget.9829 (PMC5190076; doi:10.18632/oncotarget.9829)
Supplement: Supplementary file 1 [file oncotarget-07-44023-s001.pdf]

MiR-422a promotes loco-regional recurrence by targeting NT5E/CD73 in head and neck squamous cell carcinoma

SUPPLEMENTARY FIGURES

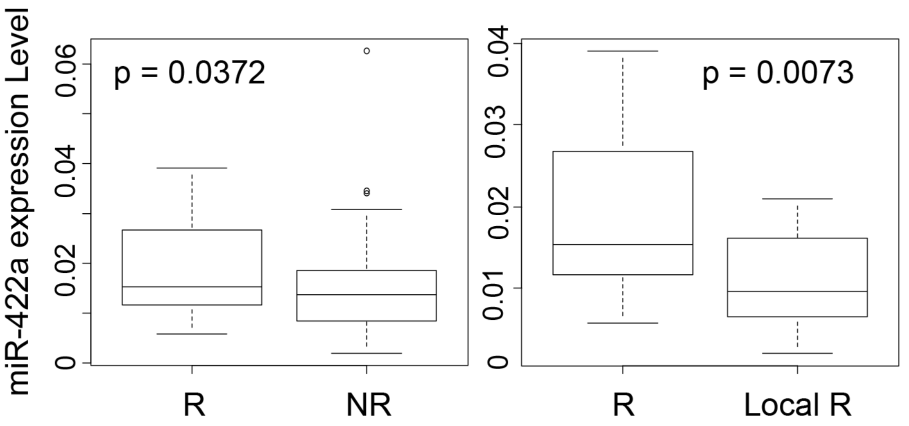

Supplementary Figure S1: Confirmation of *miR-422a* downregulation in tumors from non-responder patients using custom-made RT-qPCR. The level of expression of *miR-422a* and of the three selected reference genes: *Let.7a, miR-26a* and *Let.7b* was determined using independent custom-made RT-qPCRs. Normalized *miR-422a* expression level is significantly downregulated in tumors from non-responder patients (on the Left), particularly when they recur locally (on the Right).

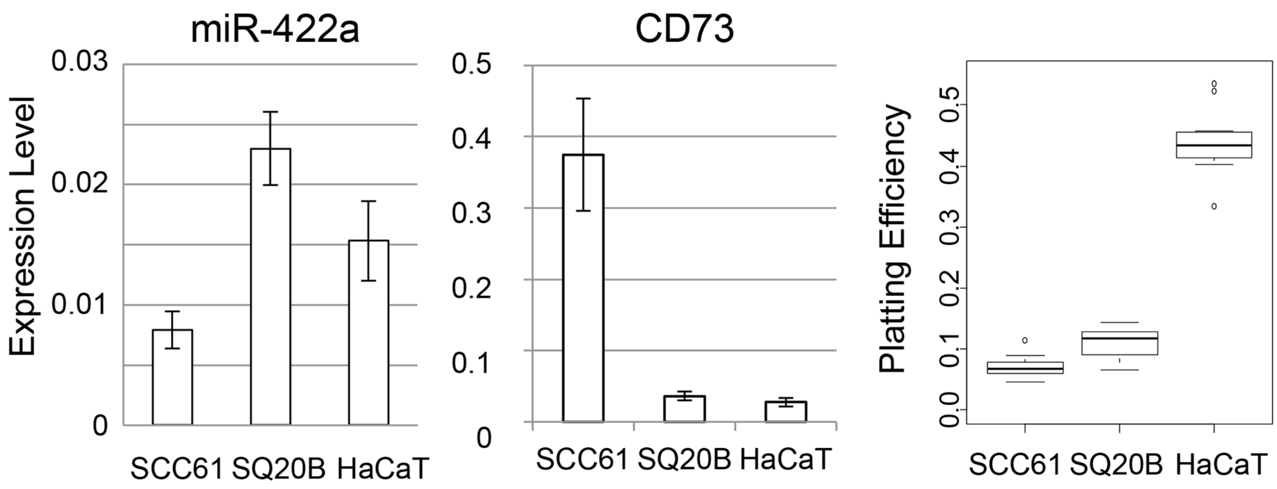

Supplementary Figure S2: Basal characteristic of SCC61, SQ20B and HaCaT cell lines. Basal expression levels of *miR-422a* (reference genes: *Let.7a, miR-26a* and *Let.7b*) (on the Left) and *CD73* (reference genes: *GAPDH, RPL19* and *TBP*) (on the middle), as well as the basal plating efficiency (on the Right), were analyzed in the three cell lines.

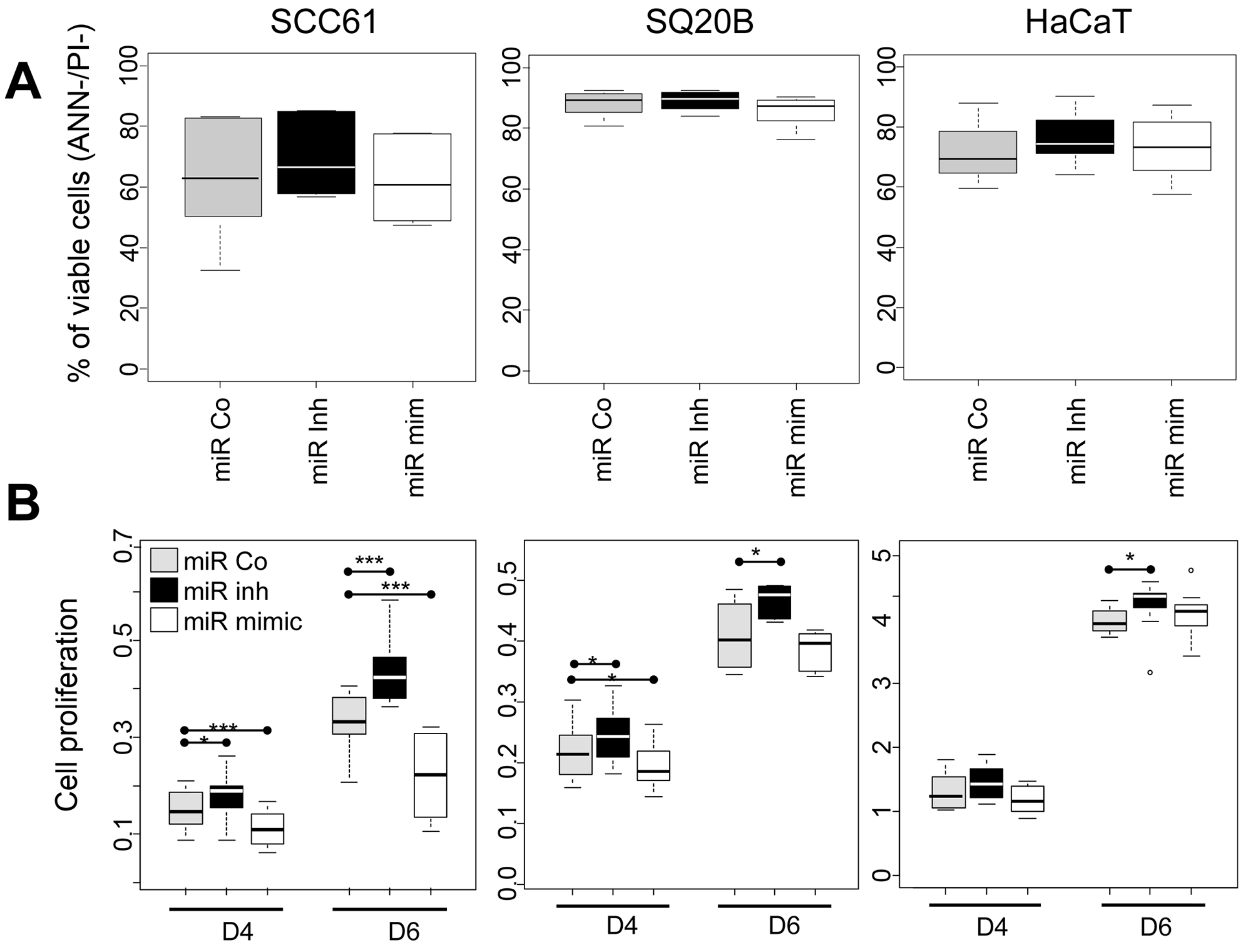

Supplementary Figure S3: *MiR-422a* modulates cell proliferation but does not influence cell viability. A. Flow cytometry determination of Annexin-V and propidium iodide (PI) negative (ANN-/IP-) population was conducted two days after transfection with the different constructs (miRCo, miRinhi, miRmim). **B.** Cells were counted using CCK8 assay at days 4 and 6 post-transfection. Luminescence Arbitrary Unit was normalized for 1 000 seeded cells. Wilcoxon tests were conducted, *p<0.05, **p<0.01, ***p<0.001.

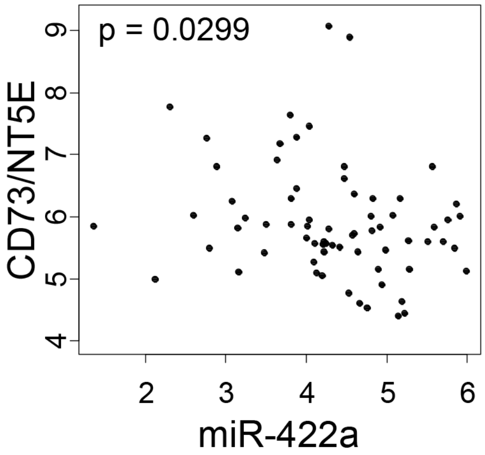

Supplementary Figure S4: *MiR-422a* and *NTE5E/CD73* expression levels are inversely correlated in an independent HNSCC cohort from the GEO dataset. Expression data of *miR-422a* and *CD73* were extracted from the GSE33232 SuperSeries of HNSCC. The *CD73* expression level is represented as a function of *miR-422a* expression (N=69 samples).

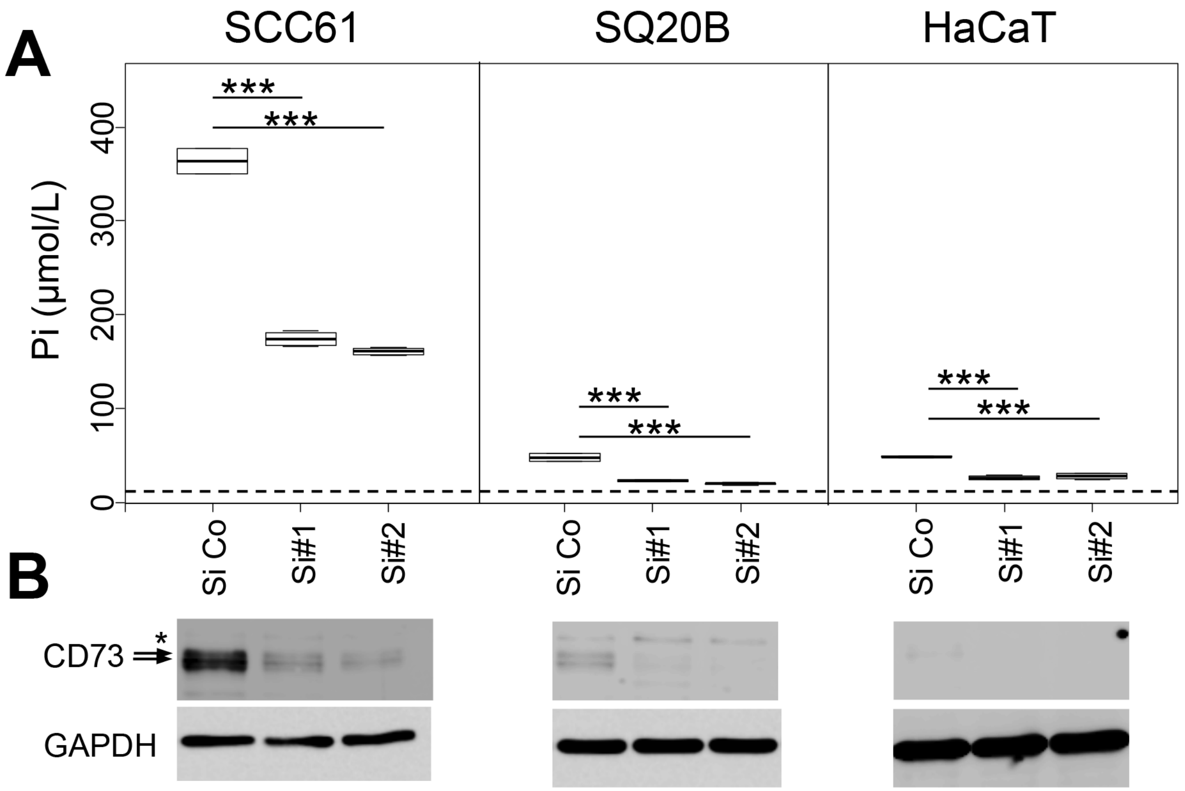

Supplementary Figure S5: Two specific siRNA efficiently inhibit *CD73* expression and activity. Cells were transfected either with an irrelevant siRNA (Si Co) or with two different siRNAs (Si#1 and Si#2) targeting *CD73*. Two days after transfection the enzymatic activity of CD73 was determined after 3h of incubation with 2mM ATP **A.** and the cellular content of CD73 was determined by Western blot analysis (20µg of lysate was used for the SCC61, 35µg for SQ20B and 70µg for HaCaT) **B**.

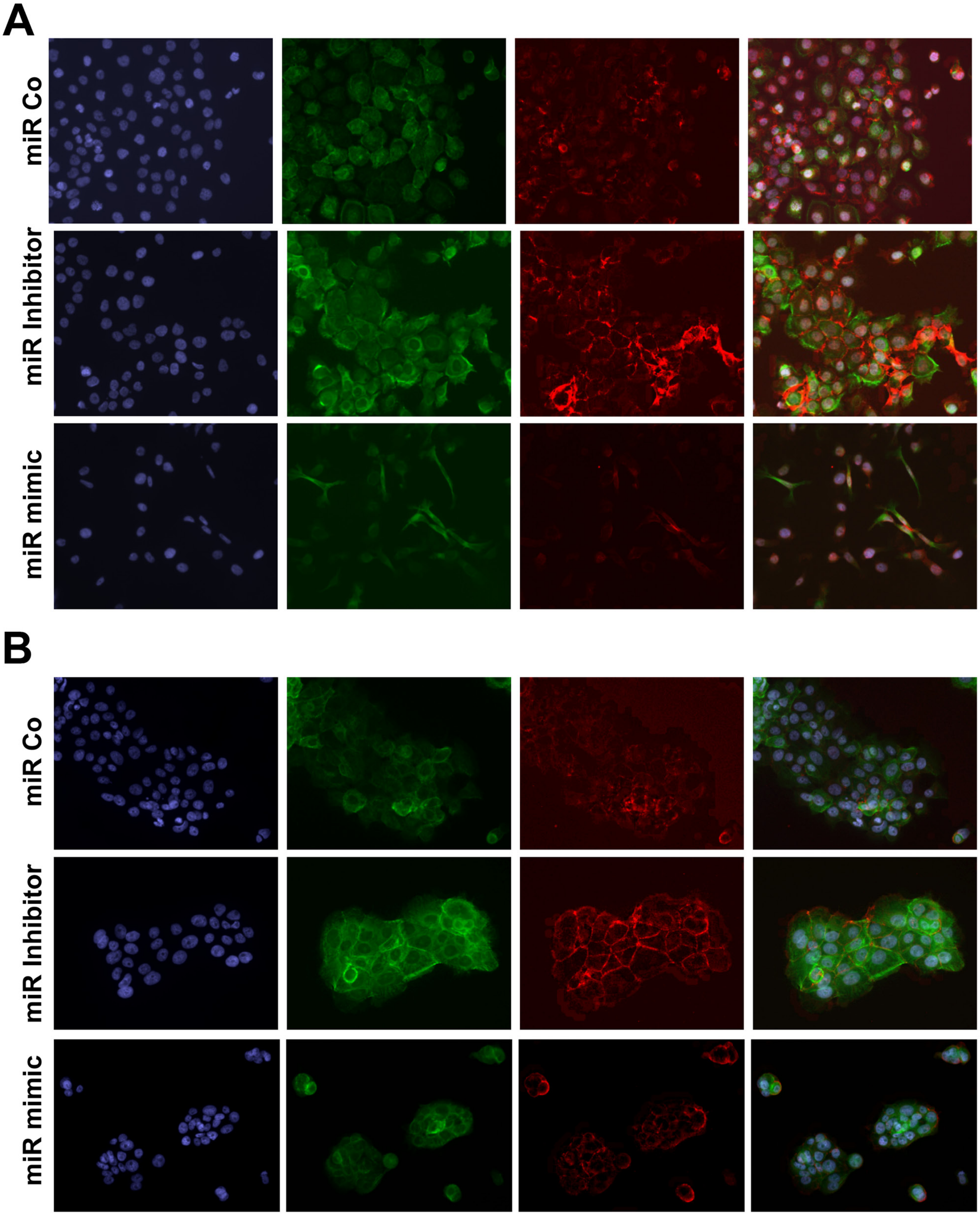

Supplementary Figure S6: Inhibition of miR-422a intensifies the recruitment of F-actin and E-cadherin at the cellular cortex. Two days after transfection of SCC61 **A.** and SQ20B **B.** cell lines, by miRmim, miRinh or miRCo, cells were fixed and labelled for nuclei (blue, first column), actin (green, second column) and E-cadherin (red, third column). An overlay is shown on the last column.

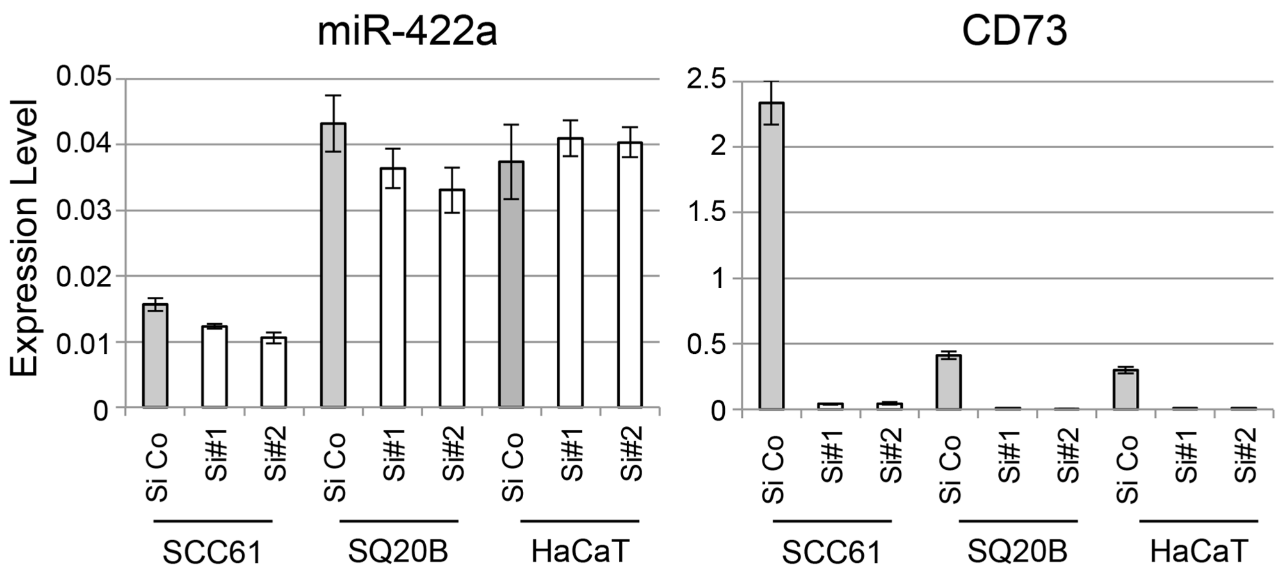

Supplementary Figure S7: CD73 knocking down only slightly impairs miR422a expression in SCC61, SQ20B and HaCaT cell lines. The level of expression of miR-422a (reference genes: *Let.7a, miR-26a* and *Let.7b*) (on the Left) and of *CD73* (reference genes: *ACTIN* and *TBP*) (on the right) was assessed two days after transfection by a control siRNA (Si Co) or two siRNA targeting CD73 (Si#1 and Si#2).

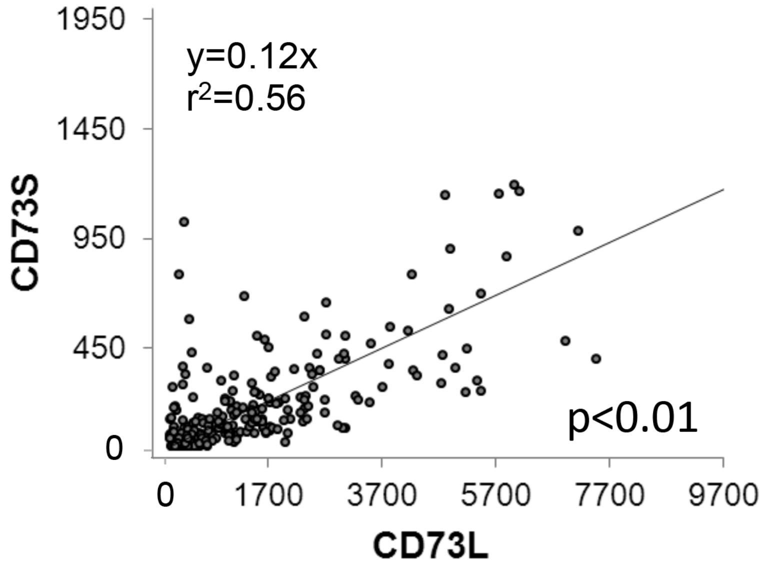

Supplementary Figure S8: The expression of the short and long isoforms of CD73 are correlated in a linear manner. The level of expression of CD73S and CD73L extracted from the TGCA cohort, are depicted. Spearman-calculated p-value is given.
